# MicroRNAs for Virus Pathogenicity and Host Responses, Identified in SARS-CoV-2 Genomes, May Play Roles in Viral-Host Co-Evolution in Putative Zoonotic Host Species

**DOI:** 10.3390/v13010117

**Published:** 2021-01-16

**Authors:** Sigrun Lange, Elif Damla Arisan, Guy H. Grant, Pinar Uysal-Onganer

**Affiliations:** 1 Tissue Architecture and Regeneration Research Group, School of Life Sciences, University of Westminster, London W1W 6UW, UK; 2 Institute of Biotechnology, Gebze Technical University, Gebze, 41400 Kocaeli, Turkey; d.arisan@gtu.edu.tr; 3 School of Life Sciences, University of Bedfordshire, Park Square, Luton LU1 3JU, UK; guy.grant@beds.ac.uk; 4 Cancer Research Group, School of Life Sciences, University of Westminster, London W1W 6UW, UK

**Keywords:** microRNA, SARS-CoV-2, COVID-19, zoonosis, co-evolution, viral pathogenesis

## Abstract

Our recent study identified seven key microRNAs (miR-8066, 5197, 3611, 3934-3p, 1307-3p, 3691-3p, 1468-5p) similar between SARS-CoV-2 and the human genome, pointing at miR-related mechanisms in viral entry and the regulatory effects on host immunity. To identify the putative roles of these miRs in zoonosis, we assessed their conservation, compared with humans, in some key wild and domestic animal carriers of zoonotic viruses, including bat, pangolin, pig, cow, rat, and chicken. Out of the seven miRs under study, miR-3611 was the most strongly conserved across all species; miR-5197 was the most conserved in pangolin, pig, cow, bat, and rat; miR-1307 was most strongly conserved in pangolin, pig, cow, bat, and human; miR-3691-3p in pangolin, cow, and human; miR-3934-3p in pig and cow, followed by pangolin and bat; miR-1468 was most conserved in pangolin, pig, and bat; while miR-8066 was most conserved in pangolin and pig. In humans, miR-3611 and miR-1307 were most conserved, while miR-8066, miR-5197, miR-3334-3p and miR-1468 were least conserved, compared with pangolin, pig, cow, and bat. Furthermore, we identified that changes in the miR-5197 nucleotides between pangolin and human can generate three new miRs, with differing tissue distribution in the brain, lung, intestines, lymph nodes, and muscle, and with different downstream regulatory effects on KEGG pathways. This may be of considerable importance as miR-5197 is localized in the spike protein transcript area of the SARS-CoV-2 genome. Our findings may indicate roles for these miRs in viral–host co-evolution in zoonotic hosts, particularly highlighting pangolin, bat, cow, and pig as putative zoonotic carriers, while highlighting the miRs’ roles in KEGG pathways linked to viral pathogenicity and host responses in humans. This in silico study paves the way for investigations into the roles of miRs in zoonotic disease.

## 1. Introduction

**Figure 1:**
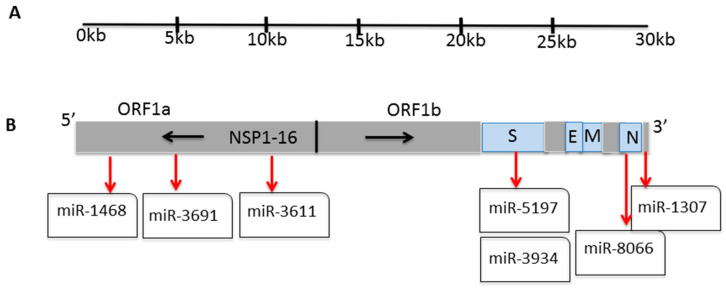
SARS-CoV-2 polycistronic genome. (**A**) The genome of SARS-CoV-2 organized in individual ORFs. (**B**) miR-1468, 3691, 3611, 5197, 3934, 8066, and 1307 locations on the SARS-CoV-2 genome (S spike; E envelope; M membrane; N nucleocapsid; NSP non-structural proteins; ORF Open reading frame).

The COVID-19 pandemic is caused by the severe acute respiratory syndrome coronavirus-2 (SARS-CoV-2), a zoonotic virus, which belongs to the betacoronavirus family. While a number of zoonotic hosts have been suggested that may possibly not show disease symptoms [1], SARS-CoV-2 causes significant pathogenicity in humans due to alterations in inflammation-related pathways, including some resulting in exacerbated inflammatory responses, vascular responses, cutaneous manifestations [2], extensive lung pathology, cardiovascular and cardiomyopathy [3,4,5], kidney damage [6,7], gastrointestinal involvement [8], as well as a wide range of neurological conditions including stroke, encephalopathy, encephalitis, central nervous system (CNS) vasculitis and acute neuropathies [9,10,11]. The SARS-CoV-2 genome contains 14 open reading frames (ORFs), preceded by transcriptional regulatory sequences (TRSs), while the two main transcriptional units, ORF1a and ORF1ab, encode replicase polyprotein 1a (PP1a) and polyprotein 1ab (PP1ab), respectively (Figure 1A). The largest polyprotein PP1ab embeds non-structural proteins (NSP1-16), which form the complex replicase machinery. This includes enzyme activities that are rare or absent in other families of positive-stranded (+) RNA viruses. The viral genomes encode four structural proteins, called spike (S), envelope (E), membrane (M), and nucleocapsid (N), and nine putative open reading frames (ORFs) for accessory factors. Non-structural proteins (NSP1-16) control an array of functions for survival for mature viruses. These vital functions include RNA-dependent polymerase (RDRp; NSP12), mRNA capping (NSPs 14 and 16), and RNA proofreading (NSP14) [12,13,14,15].

microRNAs (miRs) are short non-coding RNAs that play multifaceted roles in gene regulation, and also act as important regulators of the cellular antiviral response. Consequently, viruses have been found to utilize the host’s nuclear RNA to evade the immune response and exploit cellular machinery to their advantage by redirecting miRs to promote their replication [16]. In the last decade, miRs have not only been investigated for their diagnostic utility, but have already been applied therapeutically in different disease entities, for example, infection with hepatitis C virus (HCV) or oncological diseases [17,18]. Furthermore, miR-mediated vaccine studies showed that a number of miRs have the potential to decrease viral replication with an extensive immune response [19]. As a proof of this understanding, viral infections alter host miRs and can cause a dramatic change in host responses [19]. The aspect of the miR-mediated regulation of viral infection is though still an emerging topic, with relatively few studies so far, and therefore warrants further exploration particularly also in relation to zoonotic diseases.

Recently, we reported seven key miRs in the SARS-CoV-2 genomes that relate to host–pathogen interaction and viral pathogenicity, alongside a number of human comorbidities, and their expression was verified in miRs that are expressed in lung biopsies of SARS-CoV-2 patients and in in vitro cell models in the PRJNA615032 Bioproject trancriptome data [20]. The seven identified miRs are spread on the SARS-CoV-2 genome, with three miRs in ORF1a and four in ORF1b, whereof two are on the spike (S) protein, and two on the nuclear (N) protein (Figure 1B).

Our previous study highlighted the roles of miRs in relation to SARS-CoV-2 infection and the multifaceted symptoms associated with COVID-19 [20]. While the spread of SARS-CoV-2 from zoonotic hosts to humans has received considerable attention, investigations into the role of miRs have hitherto been limited. Zoonotic carriers suggested for SARS-CoV-2 have ranged from pangolins, bats, snakes and hedgehogs, to domestic animals such as pigs, ferrets, non-human primates as well as cats and dogs, while it is still unclear whether there is one or several carriers, and also whether the virus can jump species without the animals showing significant symptoms of illness [21]. In the vein of virus–host coevolution strategies [22,23], viruses would benefit from being in asymptomatic carriers for their own survival, but when they jump to a species which is unfamiliar with the pathogen, the new host may react with severe or unexpected immune and other host responses to the emerging pathogen, as seen for COVID-19 in humans.

In the current study, we hypothesize that miRs may play important roles in zoonosis, also forming part of virus–host coevolution. Therefore, we assessed the seven key miRs (miR-8066, 5197, 3611, 3934-3p, 1307-3p, 3691-3p, 1468-5p) previously identified by our group in the SARS-CoV-2 genome, in some of the main wild and domestic zoonotic species reported for human viruses, including suspected carriers for SARS-CoV-2, namely bat and pangolin, as well as cow, pig, chicken, and rat.

Our findings reveal differences in miR conservation between the different suspected zoonotic carriers compared with humans, indicating possible roles for these miRs in viral–host coevolution, particularly highlighting pangolin, bat, cow, and pig as putative zoonotic carriers. This is further supported by KEGG analysis of viral and pathogenic pathways linked to these miRs in humans, with particular focus on the spike associated miR-5197, which, through nucleotide differences between pangolin and human, can cause the generation of three new miRs. These display tissue specificity to brain, lung, intestine and lymph nodes, respectively, and differ in KEGG pathway regulation, possibly contributing to the adverse reaction to SARS-CoV-2 observed in the human host.

## 2. Materials and Methods

### 2.1. Genome Sequences

Genome sequences obtained from NCBI for pangolin (*Manis pentadactyla*; KN008488.1), pig (*Sus scrofa*; NC_010453.5), cow (*Bos taurus*; NC_037353.1), horseshoe bat (*Rhinolophus ferrumequinum* CM014239.1), rat (*Rattus norvegicus*; NC_005101.4), chicken (*Gallus gallus*; NC_006089.5) and human (*Homo sapiens*; NC_000004.12) were searched for similarities with the following 7 miRs: miR-8066, 5197, 3611, 3934-3p, 1307-3p, 3691-3p, 1468-5p, previously identified in the SARS-CoV-2 genome [20]. Sequence alignment was carried out using a genome-searching tool within BLASTN at NCBI. Full genome alignment of RaTG13 and SARS-CoV-2 (Wuhan-1 EPI_ISL_402125) was achieved using Clustal Omega [24] at EBI (https://www.ebi.ac.uk/Tools/msa/clustalo/).

### 2.2. Potential miR Expression and Link Analysis

The expression levels of miRs in target cells were determined by IMOTA (Interactive Multi-Omics-Tissue Atlas) [25], TissueAtlas [26], and TISSUES [27]. miRTargetLink for human [28] was used to analyze the potential link between miRs. Prediction of the RNA secondary structure in both wild type and mutated sequences for miR-5197 was analyzed by using the RNAfold database [29]. Minimum free energy (MFE) structures [30] and centroid structures [31] were calculated by the RNAfold [29].

### 2.3. Protein–Protein Network Interaction Analysis for miR Target Proteins

Search tool for the retrieval of interacting genes/proteins (STRING) analysis (https://string-db.org/) was performed on target proteins identified to be regulated by the seven miRs under study. The protein IDs were submitted and analyzed for Gene Ontology (GO) and Reactome pathways. The following parameters were applied in STRING: the functions selected were “search protein by the name”, and the chosen species database was “*Homo sapiens*”. Network analysis was further carried out by applying “basic settings” and “medium confidence”. Nodes are connected by differently colored connecting lines, which represent interactions for the network edges, based on evidence as follows: “known interactions”, which are based on experimentally determined interactions or curated databases; and “predicted interactions”, which are based on co-expression, protein homology, gene fusion, gene co-occurrence or gene neighborhood, or are established by text mining. Significant levels were considered as *p* ≤ 0.05.

## 3. Results

### 3.1. Conservation of Seven SARS-CoV-2 miRs Across Zoonotic Species and Human

**Figure 2:**
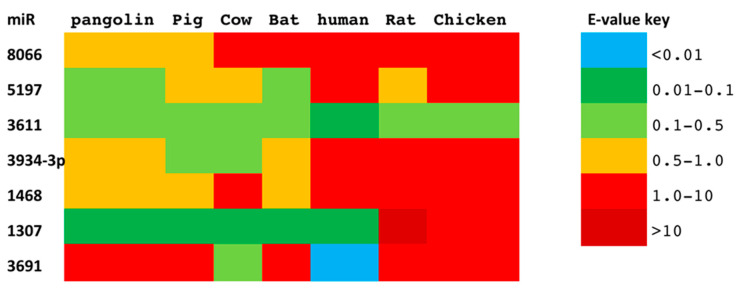
Conservation of seven SARS-CoV-2 miRs between pangolin, pig, cow, bat, rat, chicken, and human. The conservation between the seven SARS-CoV-2 miR sequences, compared with the putative zoonotic species under study and humans. Color-coding is by E-value (sequences are provided as a Supplementary Table S1).

To identify the putative conservation of our previously identified miR signature across taxa, we assessed the seven miRs in their potential source hosts, as well as in putative intermediate domestic hosts. Out of the seven miRs, miR-3611 was most strongly conserved across all species assessed; miR-5197 was most conserved in pangolin, pig, cow, bat, and rat; miR-1307 was most strongly conserved in pangolin, pig, cow, bat, and human; miR-3934-3p was most conserved in pig and cow, followed by pangolin and bat; miR-1468 was most conserved in pangolin, pig, and bat, while miR-8066 was most conserved in pangolin and pig. In human, miR-3611 and miR-1307 were most conserved, while miR-8066, miR-5197, miR-3334-3p and miR-1468 were least conserved, compared with pangolin, pig, cow, and bat (Figure 2).

### 3.2. Genomic Sequence Analysis

Using genomic sequence analysis, a high similarity was seen for the seven SARS-CoV-2 miR sequences in both bat and human as hosts (Table 1).

**Figure 3:**
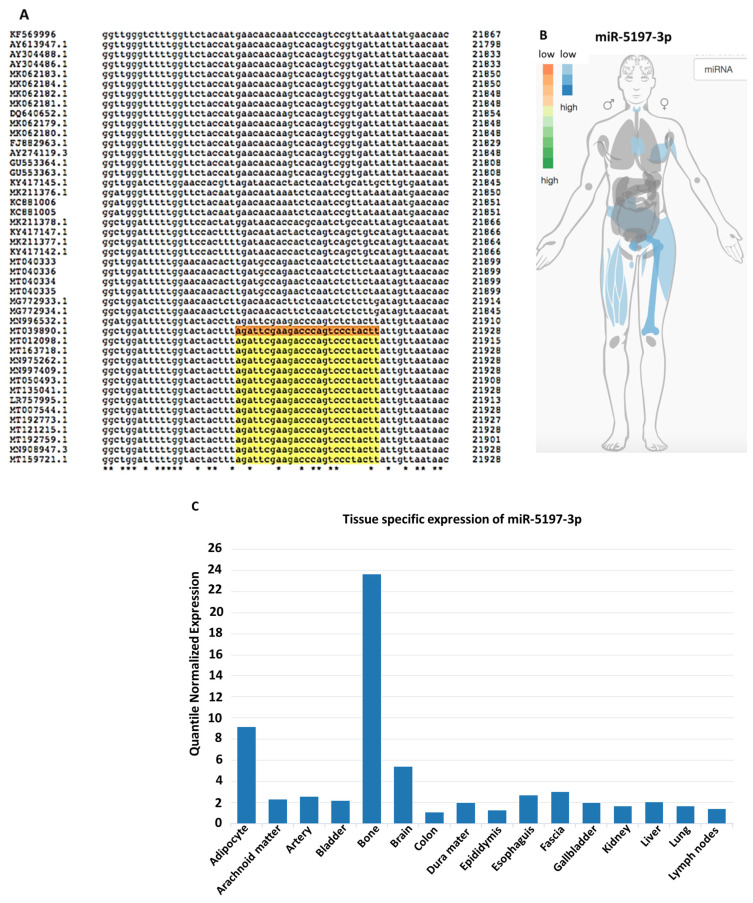

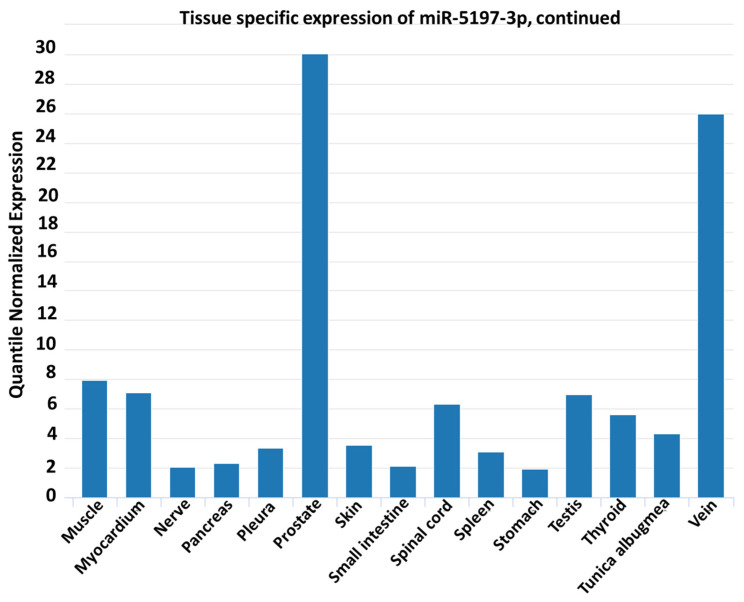
Analysis of miR-5197with respect to sequence conservation and tissue distribution. (**A**) Conservation of hsa-miR-5197 in different coronavirus samples is shown in Clustal Omega multiple sequence alignment (https://www.ebi.ac.uk/Tools/services/rest/clustalo/result/clustalo-I20200807-161825-0106-73056325-p2m/aln-clustal_num. Selected sequences were taken from Ref. [32]). (**B**) IMOTA ((Interactive Multi-Omics-Tissue Atlas) presentation of specific genes affected by miR-5197 in a tissue-specific manner. (**C**) miR-5197 expression levels and tissue distribution are shown according to tissue atlas.

**Figure 4:**
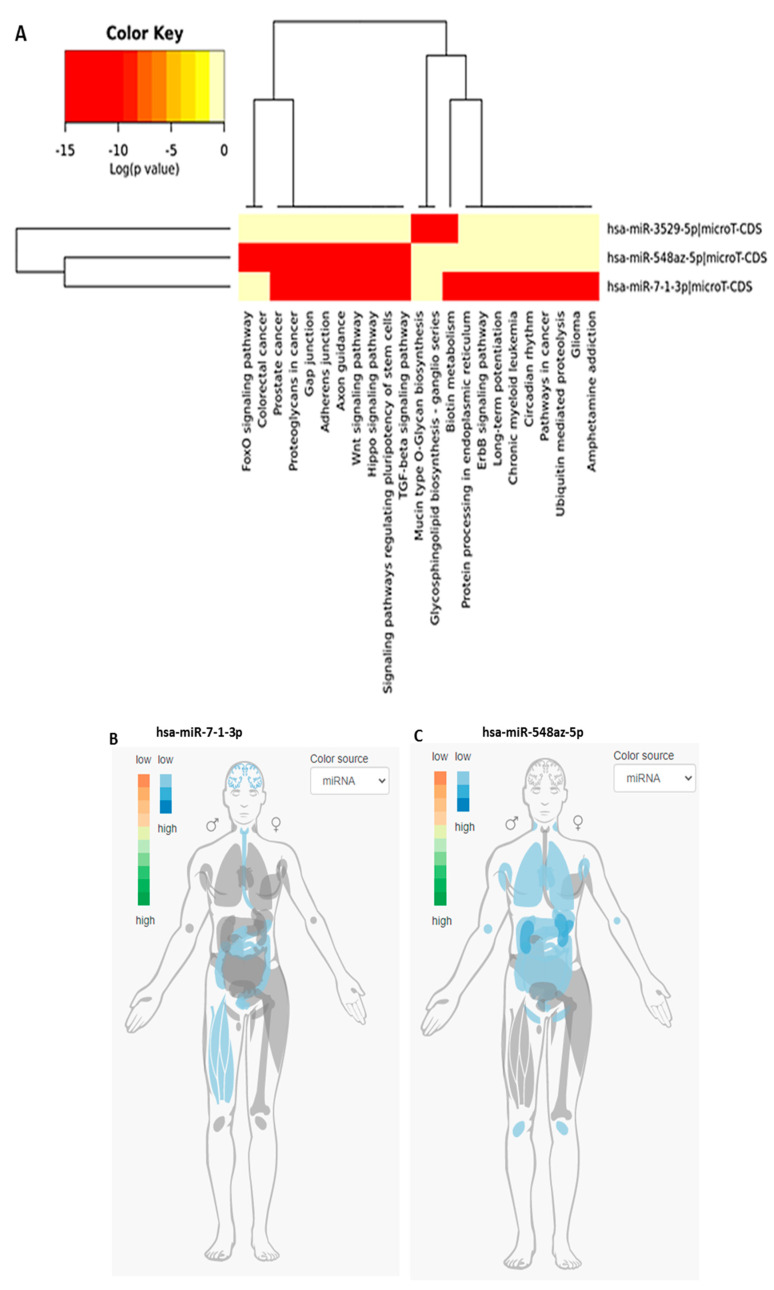

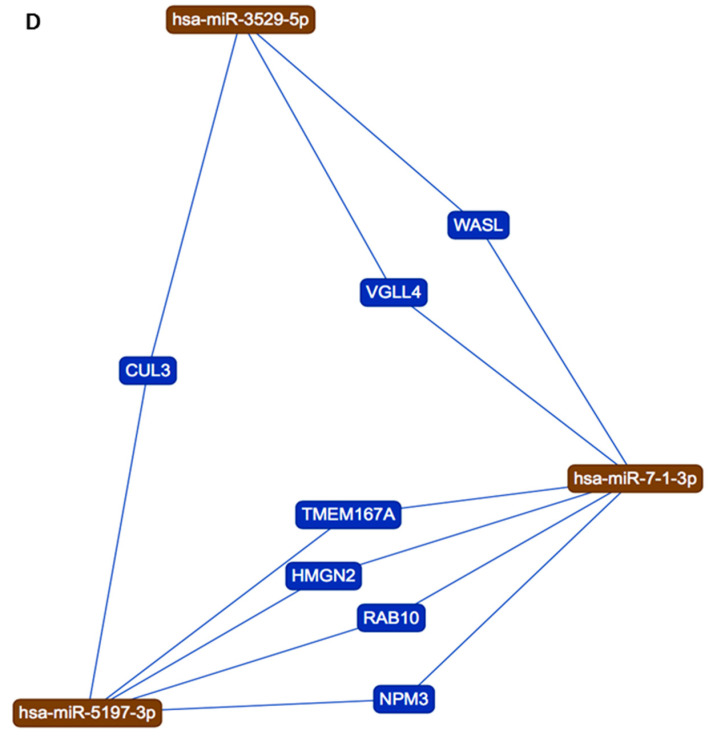
(**A**) Sequence differences between pangolin and human sequences for miR-5197 lead to the generation of three new miRs, which affect different signaling axes according to KEGG analysis. (**B**) and (**C**) IMOTA analysis for miR-7-1-3p and miR-548az-5p, respectively (IMOTA does not include miR-3529-5p); (**D**) Interaction networks with miR-5197, miR-3529-5p and miR-7-1-3p were analyzed by using miRTargetLink Human [28]. Four genes are regulated by both miR-5197-3p and miR-7-1-3p (NPM3, RAB10, HMGN2, TMEM167A), while CUL3 is regulated by both miR-5197-3p and miR-3529-5p, and VGLL4 and WASL are dependent on both miR-3529-5p and miR-7-1-3p.

While miR-5197 was conserved between bat and pangolin, it nonetheless showed higher similarity between human and bat (Table 1). Therefore, miR-5197 was further analyzed with respect to IMOTA presentation of specific genes affected by miR-5197 in a tissue-specific manner (Figure 3B), and was also assessed for tissue-specific distribution (Figure 3C). Importantly, a small change in the miR-5197 sequence in pangolin causes the predicted generation of three new miRs (miR-3529-5p, miR-7-1-3p and miR-548az-5p), which affect different KEGG signaling pathways (Figure 4). Any such change in new miR generation was not predicted for the other six miRs under study.

### 3.3. KEGG Pathway Analysis for the Three New miRs (miR-3529-5p, miR-7-1-3p, and miR-548az-5p) Generated by Changes in miR-5197

While miR-5197 is implicated in KEGG pathways for p53 signaling, cancer, and ubiquitin-mediated proteolysis (Figure S2), a range of other KEGG pathways are associated with miR-3529-5p, miR-7-1-3p, and miR-548az-5p, with a number of overlapping, but also distinctive, pathways between miR-7-1-3p and miR-548az-5p, as listed in Figure 4A, while only miR-3529-5p is associated with Mucin-O-type biosynthesis and glycosphingolipid biosynthesis (Figure 4A). When assessing the tissue distribution of miR-7-1-3p and miR-548az-5p, some differences were observed, with miR-7-1-3p predominantly being expressed in the brain, intestinal (including esophagus) tissue, and muscle (Figure 4B), but miR-548az-5p in the lung, lymph nodes, and intestines (Figure 4C). Four genes were found to be regulated by both miR-5197-3p and miR-7-1-3p (NPM3, RAB10, HMGN2, TMEM167A), while CUL3 is regulated by both miR-5197-3p and miR-3529-5p; VGLL4 and WASL are dependent on both miR-3529-5p and miR-7-1-3p. The gene interaction networks with the three miRs are shown in Figure 4D.

**Figure 5:**
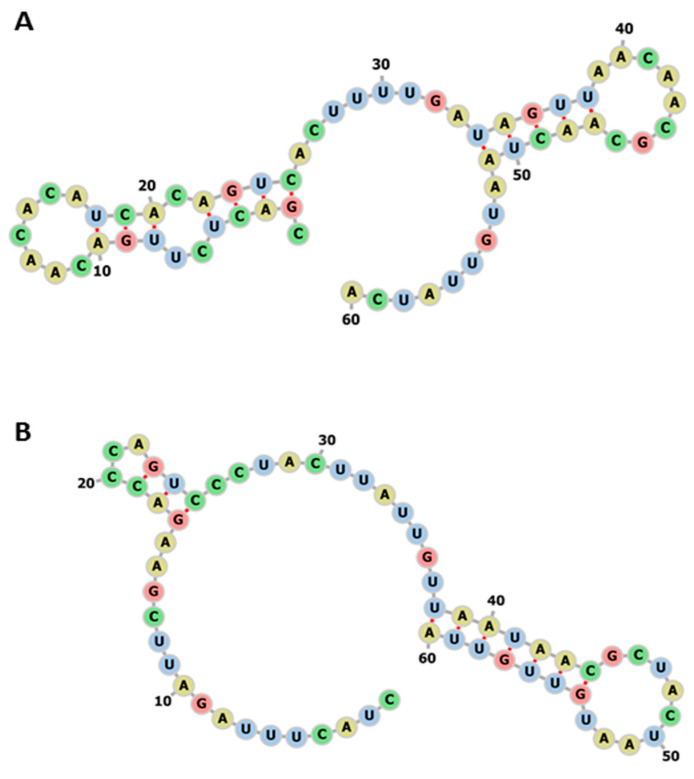
The impact of the divergence (given in Table 1) of miR-5197 in pangolin compared with human. (**A**) The RNA secondary structure of pangolin miR-5197 (MFE structure: −4.70 kcal/mol; centroid structure: −3.10 kcal/mol kcal/mol) (**B**) The RNA secondary structures of human miR-5197 (MFE structure: −3.20 kcal/mol; centroid structure: −2.90 kcal/mol) using RNAfold tool. (http://rna.tbi.univie.ac.at//cgi-bin/RNAWebSuite/RNAfold.cgi.).

The changes in miR-5197 sequences between pangolin and human affect the variations in RNA sequences, and were found to alter RNA secondary structure (Figure 5). An increase in the stability of miR-5197 in human was calculated as MFE −3.20 kcal/mol vs. −4.70 kcal/mol (centroid structure: −2.90 kcal/mol vs. −3.10 kcal/mol structures), and in pangolin as MFE (structure: −4.70 kcal/mol; centroid structure: −3.10 kcal/mol kcal/mol) according to the RNAfold tool (http://rna.tbi.univie.ac.at//cgi-bin/RNAWebSuite/RNAfold.cgi). Therefore, it can be suggested that variations between SARS-CoV-2 genomes in different hosts may possibly lead to the generation of different structural stabilities for RNA targets.

## 4. Discussion

The role of miRs in the regulation of host–pathogen interactions is still a vastly underexplored topic with significant knowledge gaps in relation to human infectious disease, including zoonosis. The current study reports in silico analysis of the conservation of seven SARS-CoV-2-specific miRs, in six species across taxa suspected to be zoonotic carriers for the virus, or previously identified as zoonotic hosts for other human viruses. Our findings indicate that these seven SARS-CoV-2-specific miRs may possibly have roles in viral–host co-evolution in zoonotic hosts, particularly highlighting pangolin, bat, cow, and pig as putative zoonotic carriers. Furthermore, these seven miRs regulate genes that play important roles in viral–host interactions and other relevant cellular and immunological processes. KEGG and GO analyses for these miRs highlighted roles linked to viral pathogenicity and host responses in humans. While these pathways may be common to numerous viral infection responses, they possibly play significant roles in SARS-CoV-2, and therefore further investigations into their specificity relating to SARS-CoV-2 infection in particular are warranted. Interestingly, we identified that changes in miR-5197 nucleotide sequences between pangolin and human can generate three predicted new miRs (miR-3529-5p, miR-7-1-3p, and miR-548az-5p), which by bioinformatics analysis show differing tissue distribution in brain, lung, intestine, lymph nodes, and muscle, and have different downstream regulatory effects on a number of KEGG pathways. This may be of considerable importance as miR-5197 is localized in the S protein transcript area of the SARS-CoV-2 genome.

From a total of 1594 and 1506 miRs from the Malayan and Chinese pangolin genomes, 333 have previously been reported using two complementary approaches *ab initio*, whereby 334 HHMMiR [33] and MiRPara [34] were shown to have similarity to the known miR335 genes in miRBase [35,36]. The miR sequences 336 accounted for <1% of the pangolin genomes, with the transposable elements-related miR-9256a-1 and miR-396c being 337 of the most abundant families [37].

Out of the seven miRs under study here, which were previously identified by us in the SARS-CoV-2 genome and verified to be expressed in lung biopsies of SARS-CoV-2 patients and in vitro cell models of infected A549 and NHEB cells [20], miR-3611 was the most strongly conserved across all species; miR-5197 was most conserved in pangolin, pig, cow, bat, and rat; miR-1307 was most strongly conserved in pangolin, pig, cow, bat, and human; miR-3691-3p in pangolin, cow, and human; miR-3934-3p was most conserved in pig and cow, followed by pangolin and bat; miR-1468 was most conserved in pangolin, pig, and bat; while miR-8066 was most conserved in pangolin and pig. In humans, miR-3611 and miR-1307 were most conserved, while miR-8066, miR-5197, miR-3934-3p and miR-1468 were least conserved, compared with pangolin, pig, cow, and bat. This indicates that pathways regulated by these different miRs may possibly play roles in host-tolerance, as well as in adverse reactions, indicating that the most conserved miRs may possibly play parts in co-evolution with the zoonotic host. On the other hand, following the virus jumping species, including into human, this may cause detrimental effects via the modulation of downstream-regulated immune and other metabolic pathways. When assessing Gene Ontology (GO) pathways for target genes and proteins affected by the different miRs, miR-3691 was linked with viral infection, viral mRNA translation, influenza life cycle, RNA processing and RNA metabolism (Figure S1). miR-5197 was related to p53 signaling, ubiquitin-mediated proteolysis, Ubl conjugation, and nucleic acid binding (Figure S2). The most conserved miR across all species, miR-3611, was associated with nucleus and cytosol (Figure S3), miR-1307 had strong links to spliceosome function and zinc finger proteins (Figure S4), miR-3934-3p to the nucleus, pre-ribosome, chromatin, and DNA pairing (Figure S5), miR-1468 to the regulation of RNA metabolism, gene expression, transcription, metabolism and the regulation of stress responses (Figure S6), and miR-8066 was related to mRNA splicing, the processing of pre-mRNA, cell cycle regulation, mTOR signaling, cell metabolism and macroautophagy (Figure S7). This indicates the differing functions for these various miRs in host–pathogen interactions, as well as in the regulation of viral transcription and cellular processing, downstream stress response regulation, and immune and metabolic functions. It can be noted that several processes listed for the various miRs above (stress response, viral infection life cycle and marcoautophagy) also link in with the interferon response [38,39], which is strongly related to viral infection [40]. Furthermore it must be considered that while some of these pathways relate generally to viral infection in the host response, the specificity of these pathways in relation to SARS-CoV-2 infection remains to be investigated in relation to strategic intervention. Therefore, in relation to other viral infections, it may also be of interest to carry out further and similar comparative analyses to aid the understanding of the disease-specific pathways mediated by miRs in different viral infections.

Interestingly, out of the seven miRs under study, miR-3691-3p was found to be the miR with the strongest association with viral infection and viral replication, as revealed by Reactome pathway analysis (Figure S1) for proteins regulated by this miR, which was found to be conserved between pangolin, cow, and human. These findings indicate that cow may be a putative intermediate host between pangolin and human, but this will require further investigation. While pangolins have been found to have unusual resistance against viral, including coronavirus, infection [41,42], the cow is also well known for its unusual antiviral responses, including those via neutralizing antibodies (“cattlebodies”), which are effective, for example, against retroviral infections such as HIV [43], and are also under investigation for their effectiveness against SARS-CoV-2. Coronaviruses are well known in cattle and can cause gastroenteritis, respiratory disease, winter dysentery and shipping fever pneumonia [44]. Furthermore, both bovine and porcine respiratory coronavirus have been shown to have features in common with both SARS-CoV and SARS-CoV-2 [45].

Of putative interest was the finding that changes in miR-5197 sequences between humans and pangolin can lead to the generation of three new miRs, which show differences in KEGG pathway regulation. The correlation to the tissue distribution of miR-7-1-3p in the brain and intestine may possibly be related to some of the COVID-19-related symptoms observed in these organs. Furthermore, based on tissue atlas analysis, miR-548az-5p is expressed in the lung, lymph nodes, and intestines, all of which are significant target organs in COVID-19. This may indicate that these miRs, due to a lack of co-evolution with the virus as is possibly observed in pangolin, may adversely affect the human host in these specific sites. This may furthermore be of importance as miR-5197 is on the spike region of the SARS-CoV-2 genome, and therefore this diversification of miR-5197 may possibly aid its regulatory activities in the distinct tissue types, consequently affecting downstream KEGG pathways. miR-5197 is critical for mucin-type O-glycan biosynthesis pathways, which relate to both human and veterinary viral infections, including HTLV-1, Ebola, HIV-1, HSV-1, avian influenza, and avian oncogenic retrovirus [46,47,48,49,50,51]. miR-5197 is also related to the KEGG morphine addiction pathway, which is linked to enhanced HIV-1 infection, HCV replicon expression, the reduced clearance of pulmonary influenza virus infection in rats, and increased SAIDS in rhesus monkeys [52,53,54,55,56,57]. miR-5197 furthermore influences the metabolism of xenobiotics by cytochrome P450 mechanisms and the TGF-β signaling pathway, which is associated with viral entry and HIV infection [58] and is strongly linked to both pulmonary and cardiovascular diseases [59,60,61]. Furthermore, miR-5197 is related to p53 regulatory pathways and cancers, ubiquitin-related proteolysis, and molecular and cellular GO pathways linked to nucleic acid binding and to nuclear and organelle function (Figure S2) (please see further in depth discussion on these pathways in Ref. [20]).

Four genes that were identified to be regulated by both miR-5197-3p and miR-7-1-3p were NPM3, RAB10, HMGN2, and TMEM167A. NPM3 has roles in ribosome biogenesis, chromatin remodeling, the protein and histone chaperone function, and the RNA-binding activity of nucleolar phosphoprotein B23/NPM [62,63]. It has furthermore been linked to lung papillary adenocarcinoma [64]. RAB10 is a small GTPase and regulates intracellular vesicle trafficking [65], and has been linked to *Legionella pneumophila* infection and replication [66]. HMGN2 binds nucleosomal DNA and is associated with transcriptionally active chromatin; it furthermore has antimicrobial activity against bacteria, viruses, and fungi [67,68], while specific roles in SARS-CoV-2 infection remain to be investigated. TMEM167A is involved in the early part of the secretory pathway, and is a regulator of vesicular trafficking [69,70]. CUL3 was identified to be regulated by both miR-5197-3p and miR-3529-5p; importantly, it has roles in endothelial cell function and angiogenesis [71], which may be of interest in relation to the strong endothelial-related responses observed in COVID-19. CUL3 also plays roles in protein homodimerization activities and ubiquitin–protein transferase activity, as well as in oxidative and electrophilic stress [13]. VGLL4 and WASL were identified to be dependent on both miR-3529-5p and miR-7-1-3p. VGLL4 is an inhibitor of cell proliferation and can act as a tumor suppressor, including via T-cell-mediated responses [72], and is also linked to meningioma and Wilson–Turner X-linked mental retardation syndrome (https://www.genecards.org/cgi-bin/carddisp.pl?gene=VGLL4). WASL regulates nuclear actin in transcriptional regulation [73], as well as actin cytoskeletal organization, including in filopodia formation and during actin remodeling for evasion strategies of NK-cell-mediated killing [74,75,76,77]. Importantly, WASL has also been found to facilitate cellular entry for a range of picornaviruses [74], while its importance in SARS-CoV-2 infection mechanisms needs verification. Diseases associated with WASL include Wiskott–Aldrich Syndrome (eczema-thrombocytopenia-immunodeficiency syndrome) [78], and WASL’s regulation of actin in the host is modified in *Mycobacterium*-mediated Buruli ulcers [79], pointing to its various roles in immune regulation and host–pathogen interactions.

Of interest, we have also noted the difference in GC content in the miR sequences between taxa in the putative zoonotic hosts, compared with human. As body temperature differs between human and these zoonotic hosts, it may be speculated that this can have some implications on miR function in the different species, as temperature is indeed an important factor for annealing, with higher GC content reflecting higher temperature tolerance. Interestingly, the higher GC content was reported to be from more stable duplexes with their targets [80]. In this context, the normal body temperature of the pangolin is 32 °C, while in bat this is 39–42 °C, the adult cow is around 38.5 °C, pig is around 38.7 °C, that of rat is 35.9 to 37.5 °C. and in adult chicken it is 40.6 °C to 41.7 °C. Previously, it has been discussed that the higher body temperature and increased metabolism in bats may serve as an evolutionary aid for their immune system by providing a powerful fight during viral infections [81]. Moreover, the stability of the miR–target interaction shows a negative correlation with body temperature; in other words, lower GC contents at higher body temperatures result in less functional stability in miR–mRNA interactions [82,83]. Furthermore, as the calculated stability of miR-5197 in the human differed from that of pangolin, this may suggest that possible variations between SARS-CoV-2 genomes in different hosts may lead to the generation of different structural stabilities in RNA targets.

Indeed, various zoonotic hosts for SARS-CoV-2 have been discussed in the literature during the current COVID-19 pandemic, but also in relation to the previous SARS-CoV outbreaks. This has pointed to the involvement of, for example, bats, snakes, and pangolins, but also, as many viruses jump from wild to domestic animals, sometimes having multiple hosts, due to the disruption of ecological balance and habitat shifts caused by anthropogenic activities, there are a number of domestic and companion animals that may need to be considered, particularly as these will be in close contact with humans [84,85]. As SARS-CoV-2 has indeed been reported in domestic species, such as felines and canines, we have in the current study, besides bat and pangolin, also assessed the seven miRs in cow, pig, chicken, and rat as exemplar domestic species, which have historically been linked to a range of zoonotic diseases. In this context, the recent SARS-CoV-2 transmission to mink on Danish mink farms further emphasizes the adaptability of this zoonotic virus, and the risk of species-jumping and transmission caused by anthropogenic changes.

The aspect of miR-mediated regulation in viral infection is an emerging topic, with relatively few studies so far in relation to human host responses, and it therefore warrants further exploration, particularity also in relation to zoonotic disease, including in wild and domestic species. Therefore, our identification here of differences in the conservation of the seven SARS-CoV-2-specific miRs previously identified in human in the several candidate zoonotic carriers under study, compared with human, may contribute to furthering understanding of the miR-mediated regulation of virus–host coevolution, and its roles in zoonotic disease spread and tolerance between species. Such miR-mediated regulation also may help to further understand some of the detrimental effects observed in human host immune responses when encountering new zoonotic pathogens.

With this in silico study we hope to pave the way for furthering research into the regulatory roles of miRs in zoonosis. Targeting miRs in emerging infectious diseases may be a promising novel strategy for therapeutic intervention.

## 5. Conclusions

The role of microRNAs in the regulation of host–pathogen interactions is a vastly underexplored topic with significant knowledge gaps in relation to human infectious disease, including zoonosis. The current study reports the conservation of seven SARS-CoV-2-specific microRNAs in six species across taxa suspected to be zoonotic carriers for the virus, or previously identified as zoonotic hosts for other human viruses. Our in silico analysis indicates that these SARS-CoV-2-specific miRs may play possible roles in viral–host co-evolution in a number of zoonotic hosts, particularly highlighting pangolin, bat, cow, and pig as putative zoonotic carriers. Our findings may contribute to the current understanding of some of the detrimental effects observed by human host immune responses when encountering new zoonotic pathogens, and pave the way for further investigations into the roles of miRs in zoonosis. Targeting miRs in emerging infectious diseases may be a promising strategy for novel therapeutic intervention, which warrants further investigation.

## Figures and Tables

**Table 1:** Comparison of previously identified human miR sequences [20] in SARS-CoV-2 (human) with pangolin and bat coronaviruses. Segments extracted from multiple sequence alignment of complete viral genomes. Human miR sequences are identified in red; sequence differences in the other species are in green. GC content (%) and animal body temperature are also included in the table.

miR	miR Sequence Alignment			GC Content(%)	Body Temperature (°C)
8066	ATATGGGTTGCAAATGAGGGAGCCTTGAATACACCTAAAGATCACATTGGCACCCGAAA	28677	Pangolin	31	32
	ATATGGGTTGCAACTGAGGGAGCCTTGAATACACCAAAAGATCACATTGGCACCCGCAAT	28723	Human	37.5	37
	ATATGGGTTGCAACTGAGGGAGCCTTGAATACACCAAAAGATCACATTGGCACCCGCAAT	28689	Bat	37.5	39–42
5197	CGACTCTTGACAACACATCACAGTCACTTTTGATAGTTAACAACGCAACTAATGTTATCA	21922	Pangolin	40	32
	CTACTTTAGATTCGAAGACCCAGTCCCTACTTATTGTTAATAACGCTACTAATGTTGTTA	21944	Human	48	37
	CTACCTTAGATTCGAAGACCCAGTCTCTACTTATTGTTAATAACGCTACTAATGTTGTTA	21926	Bat	43	39–42
3611	CAAGAGCGCTTTTTACATACTACCATCCATTGTCTCTAATGAGAAAGAAGAAATTCTTGG	4350	Pangolin	25	32
	TAAAAGTGCCTTTTACATTCTACCATCTATTATCTCTAATGAGAAGCAAGAAATTCTTGG	4371	Human	31	37
	TAAAAGTGCCTTTTACATTCTACCATCTATTATCTCTAATGAGAAGCAAGAAATTCTTGG	4353	Bat	31	39–42
3934	GACCCCATGCCTAATAAT---------GGCTGGACAGTCTTTTCAGCTGCTTATTACGTG	22329	Pangolin		32
	TATTTGACTCCTGGTGATTCTTCTTCAGGTTGGACAGCTGGTGCTGCAGCTTATTATGTG	22363	Human	42	37
	TATTTGACTCCTGGTGATTCTTCTTCAGGTTGGACAGCTGGTGCTGCAGCTTATTATGTG	22345	Bat	42	39–42
1468	ACACGTCCAACTCAGTTTGCCTGTTTTACAGGTTCGCGACGTGCTCGTACGTGGCTTTGG	360	Pangolin	42	32
	ACACGTCCAACTCAGTTTGCCTGTTTTACAGGTTCGCGACGTGCTCGTACGTGGCTTTGG	360	Human	42	37
	ACACGTCCAACTCAGTTTGCCTGTCTTACAGGTTCGCGACGTGCTCGTACGTGGCTTTGG	345	Bat	50	39–42
1307	TGTGTAACATTAGGGAGGACTTGAAAGAGCCACCACATTTTCACCGAGGCCACGCGGAGT	29702	Pangolin	76	32
	TGTGTAACATTAGGGAGGACTTGAAAGAGCCACCACATTTTCACCGAGGCCACGCGGAGT	29748	Human	76	37
	TGTGTAACATTAGGGAGGACTTGAAAGAGCCACCACATTTTCACCGAGGCCACGCGGAGT	29714	Bat	76	39–42
3691	GAGATGTTGATACAGACTTTGTGAATGAGTTTTATGCATATTTGCGTAAACACTTCTCAA	15682	Pangolin	36	32
	GAGATGTTGACACAGACTTTGTGAATGAGTTTTACGCATATTTGCGTAAACATTTCTCAA	15703	Human	41	37
	GAGATGTTGACACAGACTTTGTGAATGAGTTTTACGCATATTTGCGTAAACATTTCTCAA	15685	Bat	41	39–42

## Data Availability

Publicly available datasets were analyzed in this study. The NCBI data access numbers, with the exception of Wuhan one (Wuhan-1 EPI_ISL_402125) which was obtained from GISEAD, referred to accordingly in the text and can be found here: KN008488.1; NC_010453.5; NC_037353.1; CM014239.1; NC_005101.4; NC_006089.5; NC_000004.12; Wuhan-1 EPI_ISL_402125.

## Supplementary Materials

The following are available online at https://www.mdpi.com/1999-4915/13/1/117/s1, Figure S1, Reactome pathways for miR-3691-regulated target proteins. Target genes for miR-3691 were identified using miR base and interaction networks generated using STRING analysis. Reactome pathways (A,B), KEGG pathways (C), UniProt keywords (D) and molecular function GO pathways (E) are highlighted in the different color nodes, see color key in the figure. Known and predicted interactions are indicated by differently colored lines (see color key in figure). Figure S2, Interaction pathways for miR-5197-regulated target proteins. Target genes for miR-5197 were identified using miR base and interaction networks generated using STRING analysis. The following pathways are highlighted: (A) KEGG pathways; (B) UniProt keywords; (C) Molecular GO function; (D) Cellular GO pathways. The individual pathways are highlighted in the different color nodes, see color key in the figure. Known and predicted interactions are indicated by differently colored lines (see color key in figure). Figure S3, Interaction pathways for miR-3611-regulated target proteins. Target genes for miR-3611 were identified using miR base and interaction networks generated using STRING analysis. The following pathways are highlighted: (A) Cellular GO component; (B) Biological GO process. The individual pathways are highlighted in the different color nodes, see color key in the figure. Known and predicted interactions are indicated by differently colored lines (see color key in figure). Figure S4, Interaction pathways for miR-1307-regulated target proteins. Target genes for miR-1307 were identified using miR base and interaction networks generated using STRING analysis. The following pathways are highlighted: (A) Cellular GO component; (B) PFAM protein domains; (C) INTERPRO protein domains; (D) SMART protein domains. The individual pathways are highlighted in the different color nodes, see color key in the figure. Known and predicted interactions are indicated by differently colored lines (see color key in figure). Figure S5, Interaction pathways for miR-3934-regulated target proteins. Target genes for miR-1307 were identified using miR base and interaction networks generated using STRING analysis. The following pathways are highlighted: (A) Cellular GO component; (B) Reactome pathways. The individual pathways are highlighted in the different color nodes, see color key in the figure. Known and predicted interactions are indicated by differently colored lines (see color key in figure). Figure S6, Interaction pathways for miR-1468-regulated target proteins. Target genes for miR-1468 were identified using miR base and interaction networks generated using STRING analysis. The following pathways are highlighted: (A) Biological GO process; (B) Cellular GO component. The individual pathways are highlighted in the different color nodes, see color key in the figure. Known and predicted interactions are indicated by differently colored lines (see color key in figure). Figure S7, Interaction pathways for miR-8066-regulated target proteins. Target genes for miR-1468 were identified using miR base and interaction networks generated using STRING analysis. The following pathways are highlighted: (A) Reactome pathways; (B) Biological GO process; (C) Cellular GO component. The individual pathways are highlighted in the different color nodes, see color key in the figure. Known and predicted interactions are indicated by differently colored lines (see color key in figure). Table S1, The conservation between the seven SARS-CoV-2 miR sequences, compared with the putative zoonotic species under study and humans.
