# Supplementary figures and images for: MicroRNAs for Virus Pathogenicity and Host Responses, Identified in SARS-CoV-2 Genomes, May Play Roles in Viral-Host Co-Evolution in Putative Zoonotic Host Species
Source: Viruses. 2021 Jan 16;13(1):117. doi: 10.3390/v13010117 (PMC7830670; doi:10.3390/v13010117)

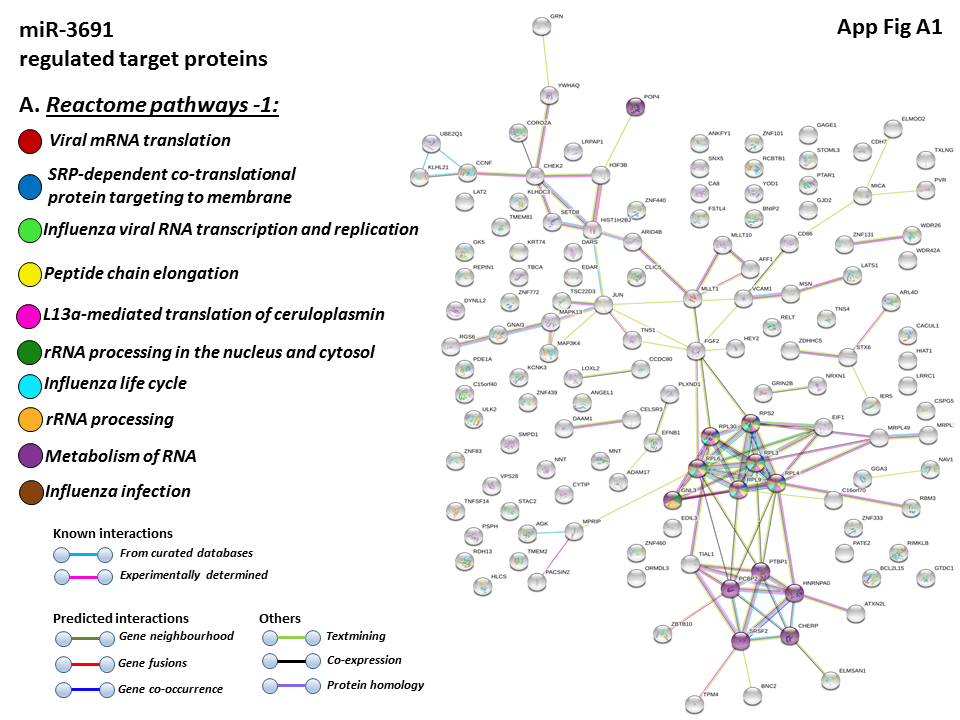

Supplement: Supplementary file 1 [file viruses-13-00117-s001.zip › Supp Fig S1_A_miR Zoonosis_2020.tif]

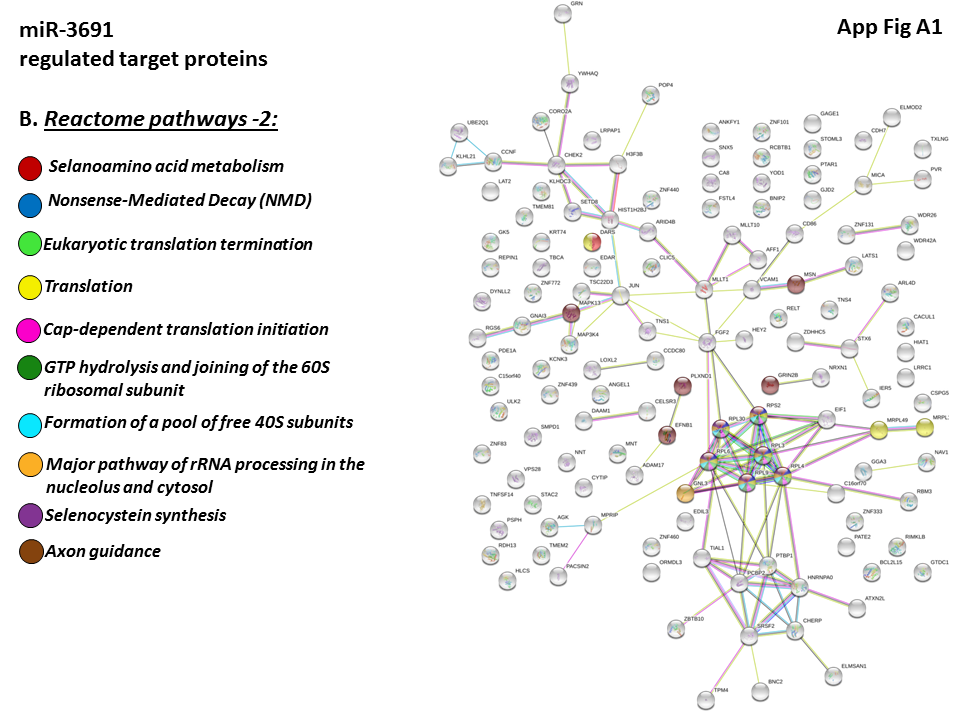

Supplement: Supplementary file 1 [file viruses-13-00117-s001.zip › Supp Fig S1_B_miR Zoonosis_2020.tif]

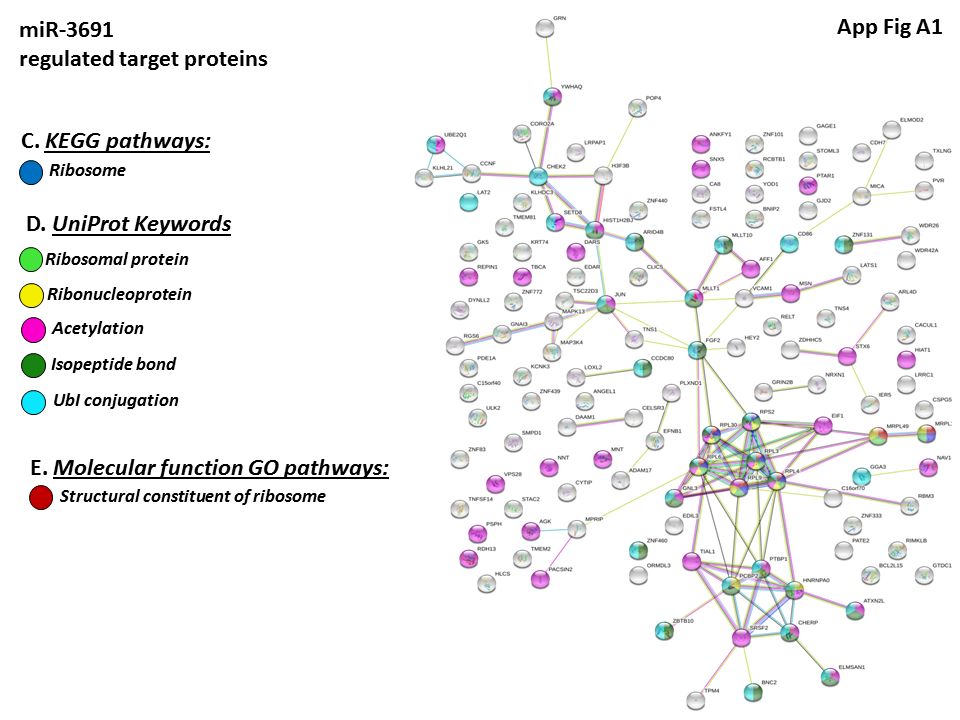

Supplement: Supplementary file 1 [file viruses-13-00117-s001.zip › Supp Fig S1_C-E_miR Zoonosis_2020.tif]

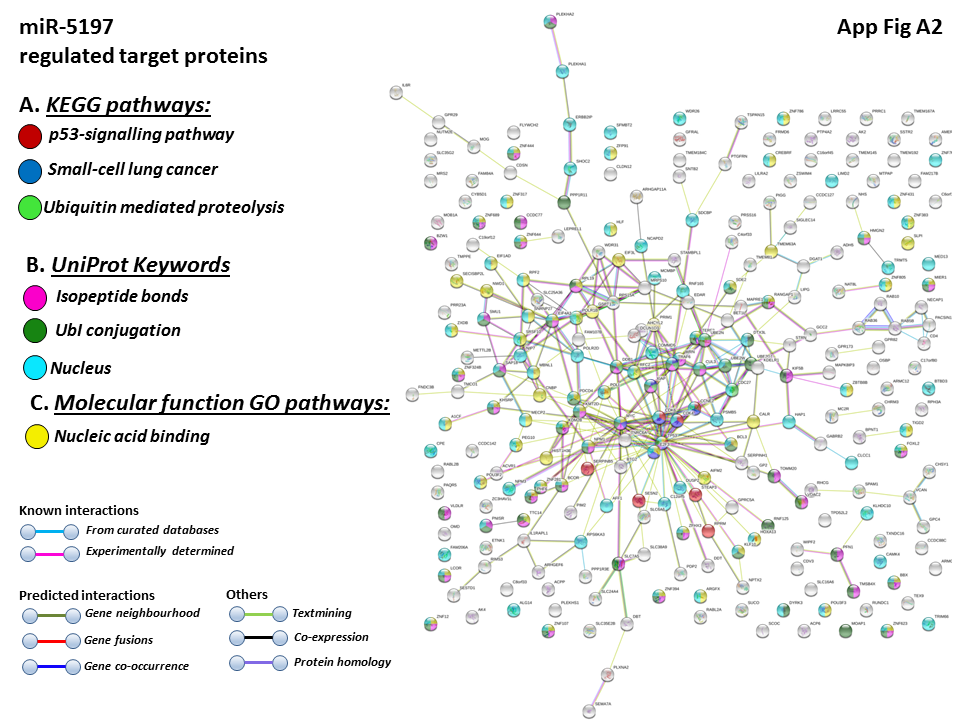

Supplement: Supplementary file 1 [file viruses-13-00117-s001.zip › Supp Fig S2_A-C_miR Zoonosis_2020.tif]

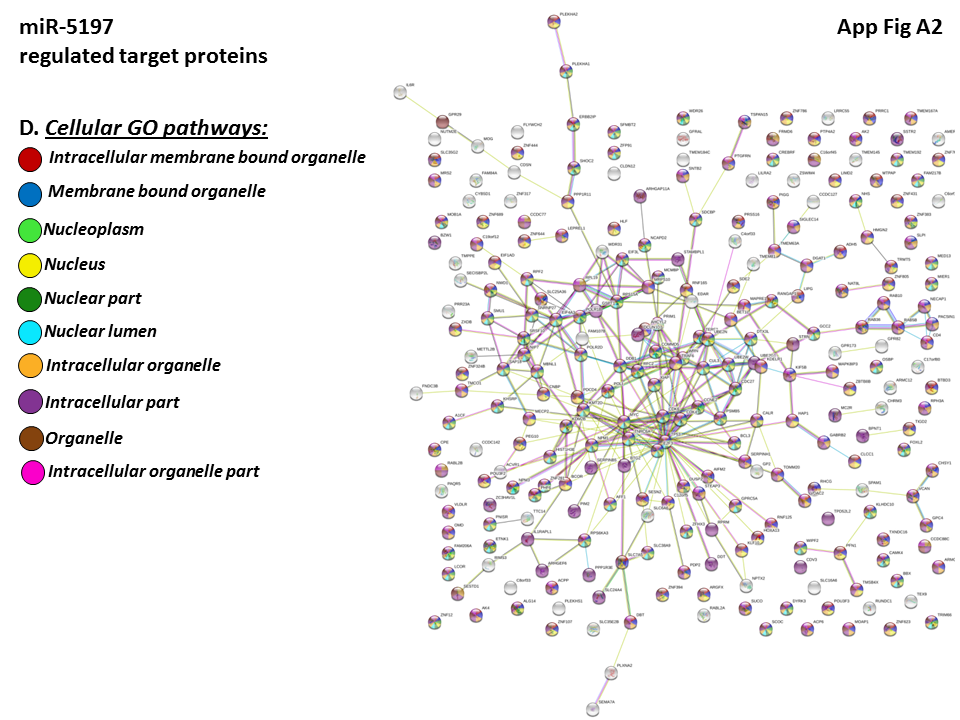

Supplement: Supplementary file 1 [file viruses-13-00117-s001.zip › Supp Fig S2_D_miR Zoonosis_2020.tif]

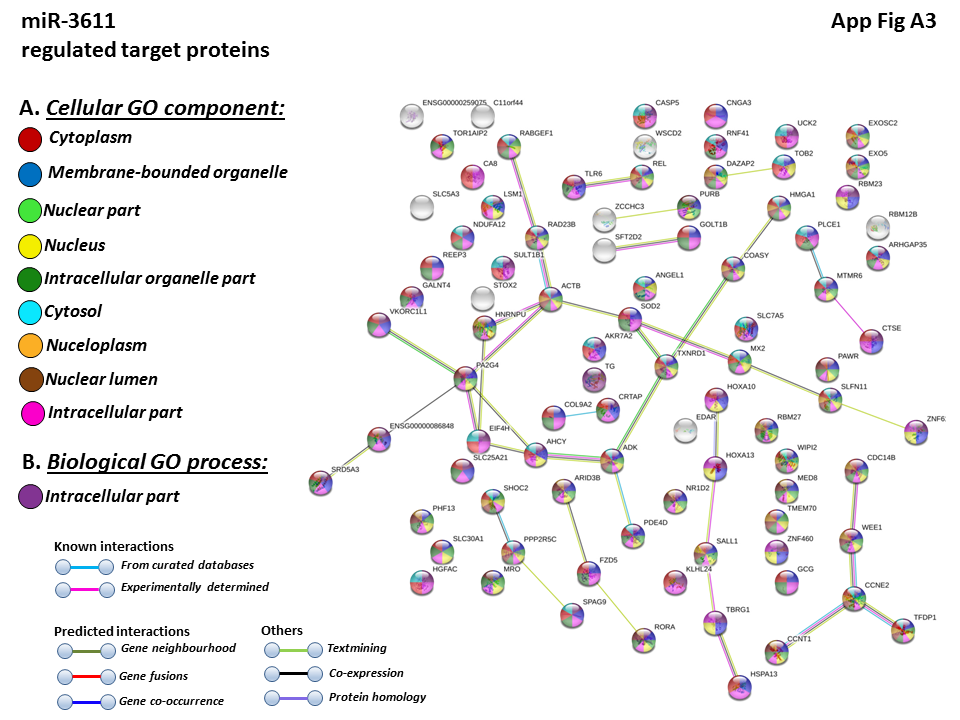

Supplement: Supplementary file 1 [file viruses-13-00117-s001.zip › Supp Fig S3_miR Zoonosis_2020.tif]

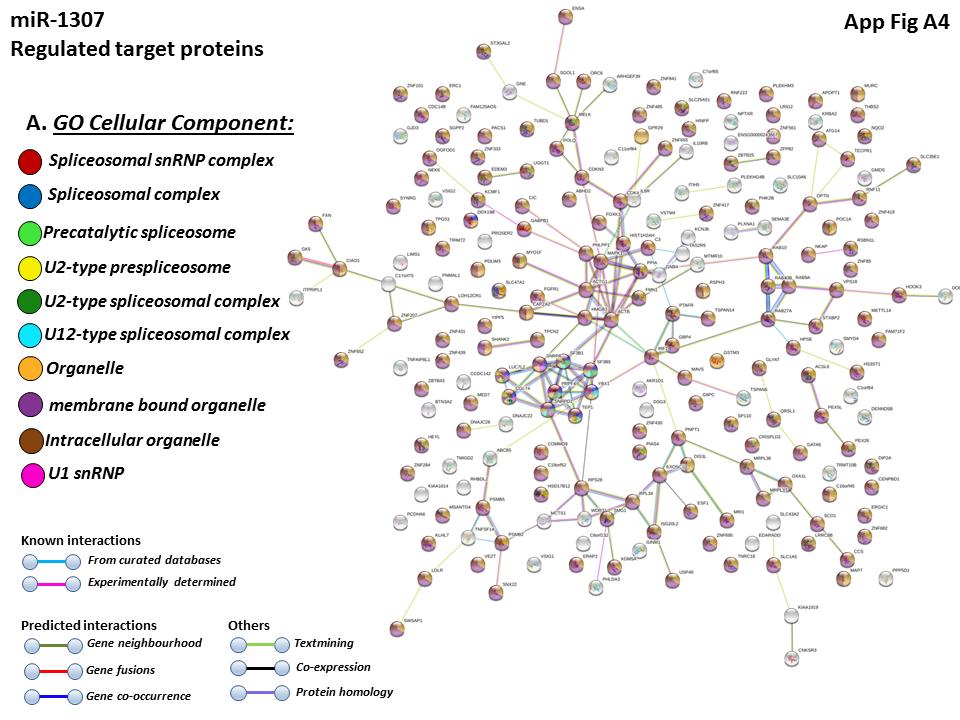

Supplement: Supplementary file 1 [file viruses-13-00117-s001.zip › Supp Fig S4_A_miR Zoonosis_2020.tif]

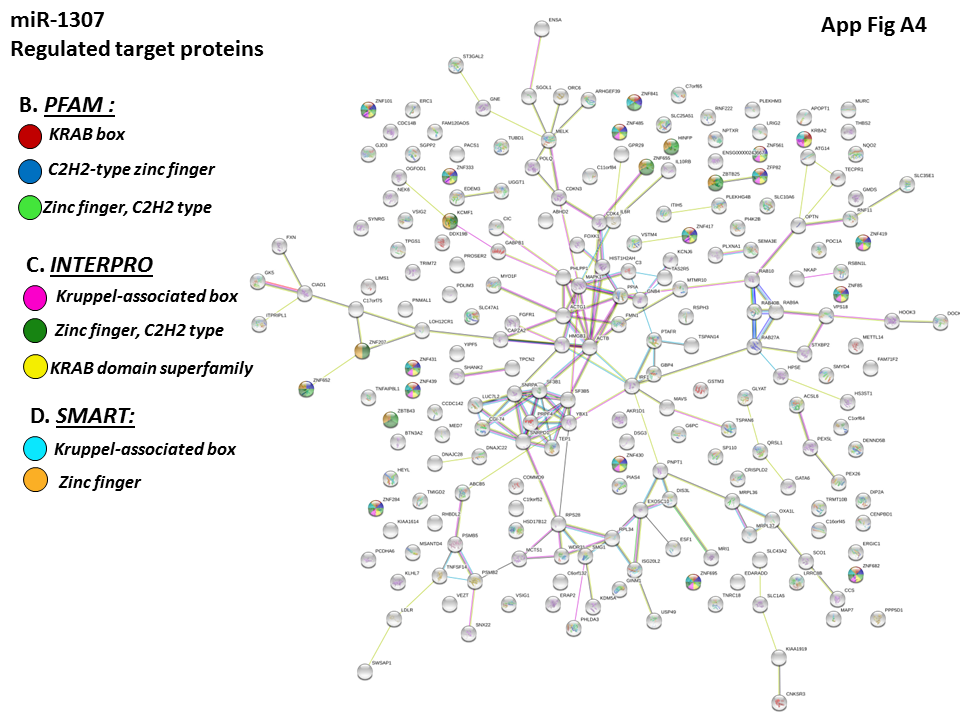

Supplement: Supplementary file 1 [file viruses-13-00117-s001.zip › Supp Fig S4_B-D_miR Zoonosis_2020.tif]

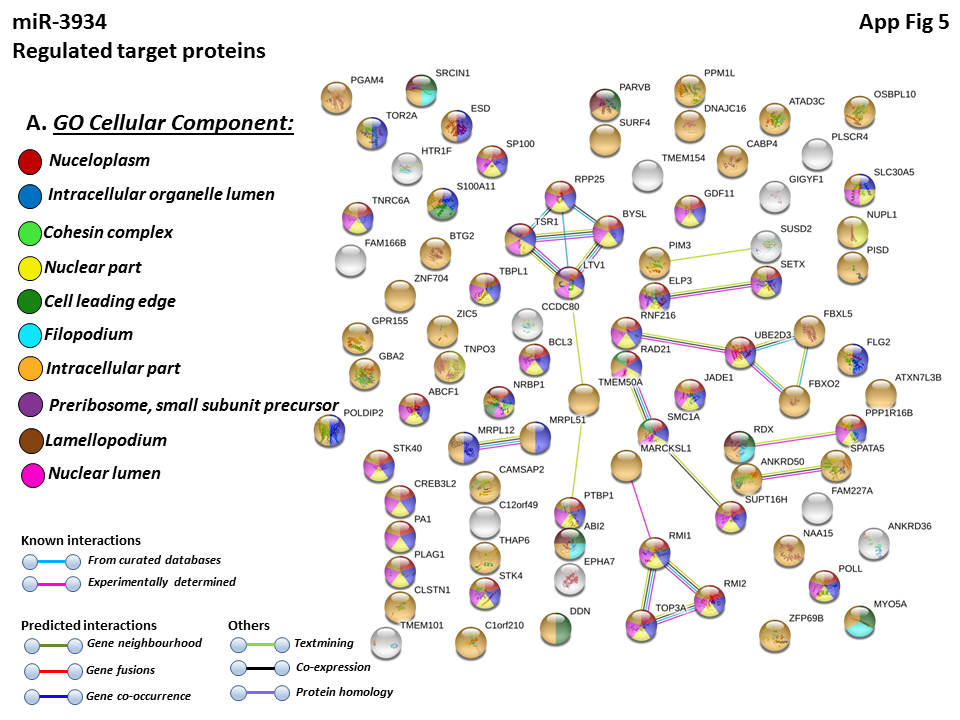

Supplement: Supplementary file 1 [file viruses-13-00117-s001.zip › Supp Fig S5_A_miR Zoonosis_2020.tif]

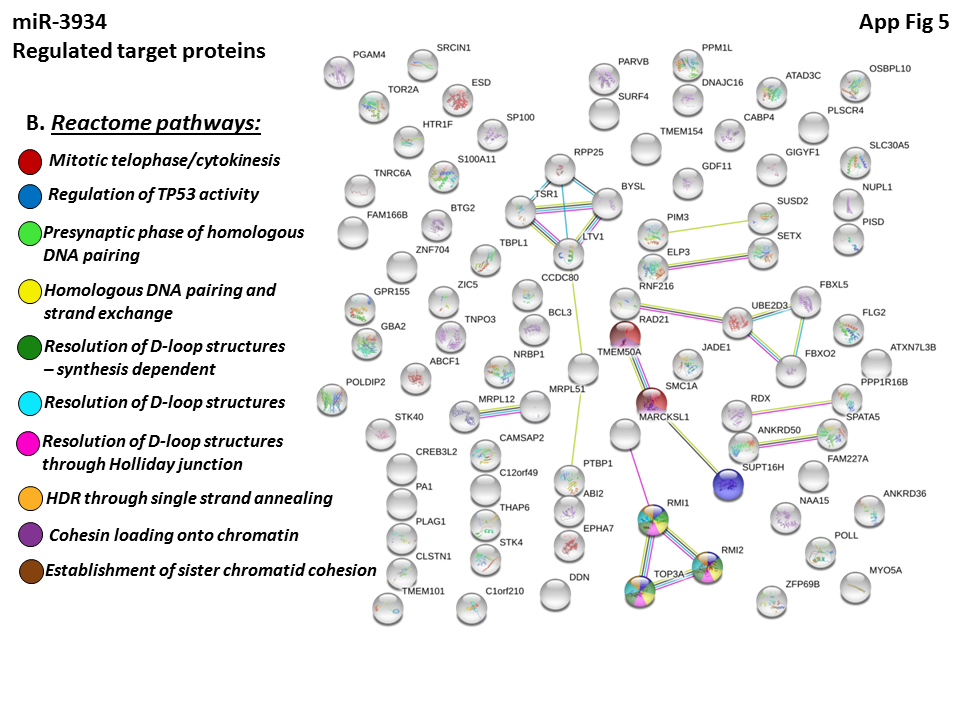

Supplement: Supplementary file 1 [file viruses-13-00117-s001.zip › Supp Fig S5_B_miR Zoonosis_2020.tif]

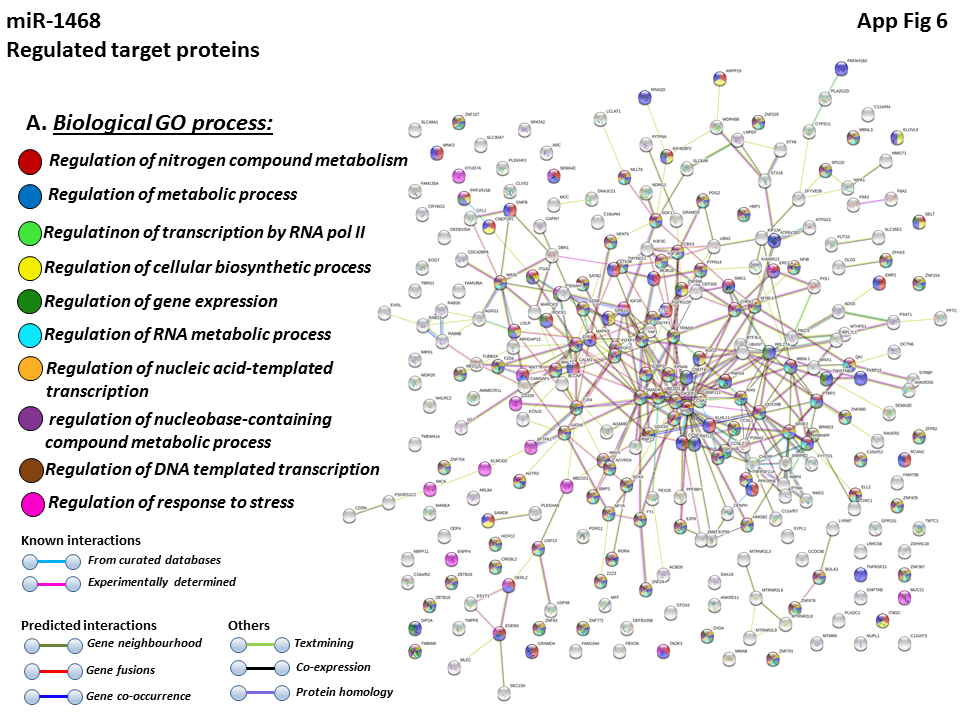

Supplement: Supplementary file 1 [file viruses-13-00117-s001.zip › Supp Fig S6_A_miR Zoonosis_2020.tif]

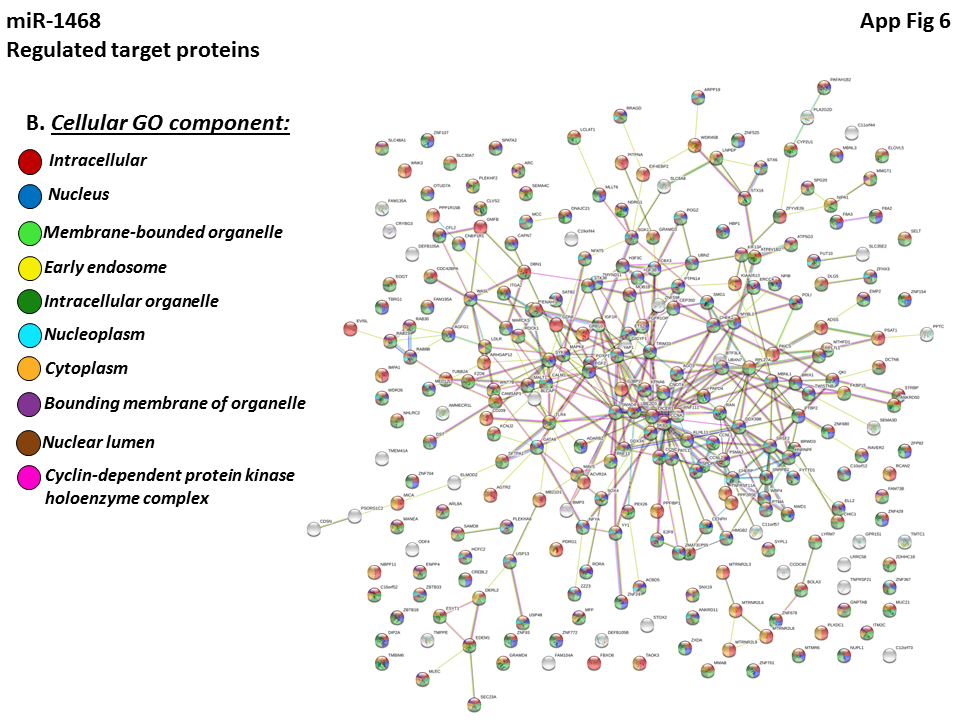

Supplement: Supplementary file 1 [file viruses-13-00117-s001.zip › Supp Fig S6_B_miR Zoonosis_2020.tif]

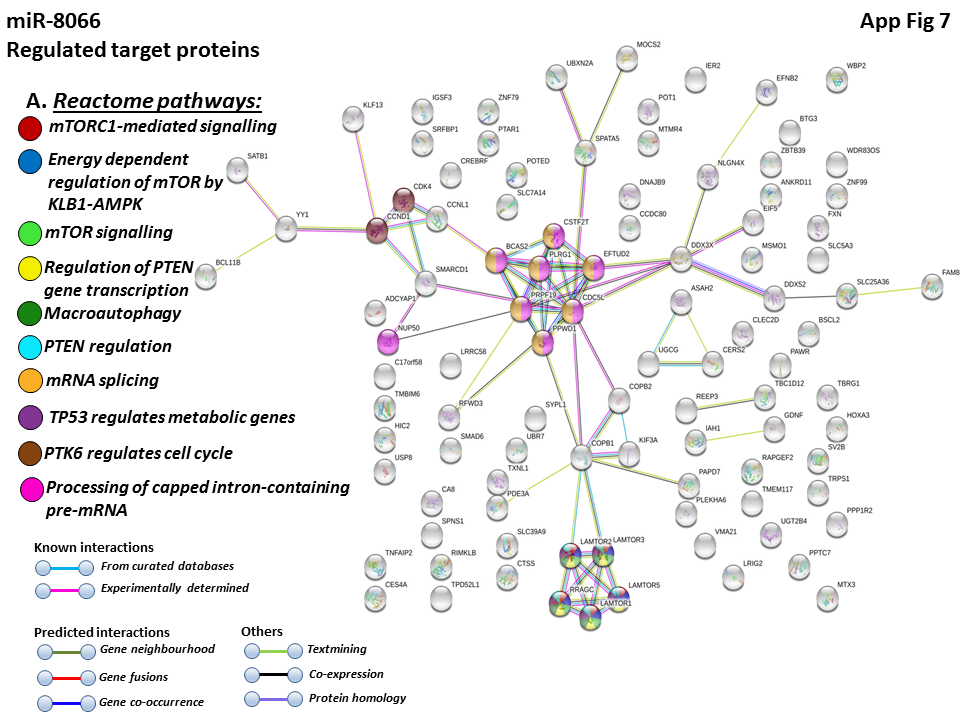

Supplement: Supplementary file 1 [file viruses-13-00117-s001.zip › Supp Fig S7_A_miR Zoonosis_2020.tif]

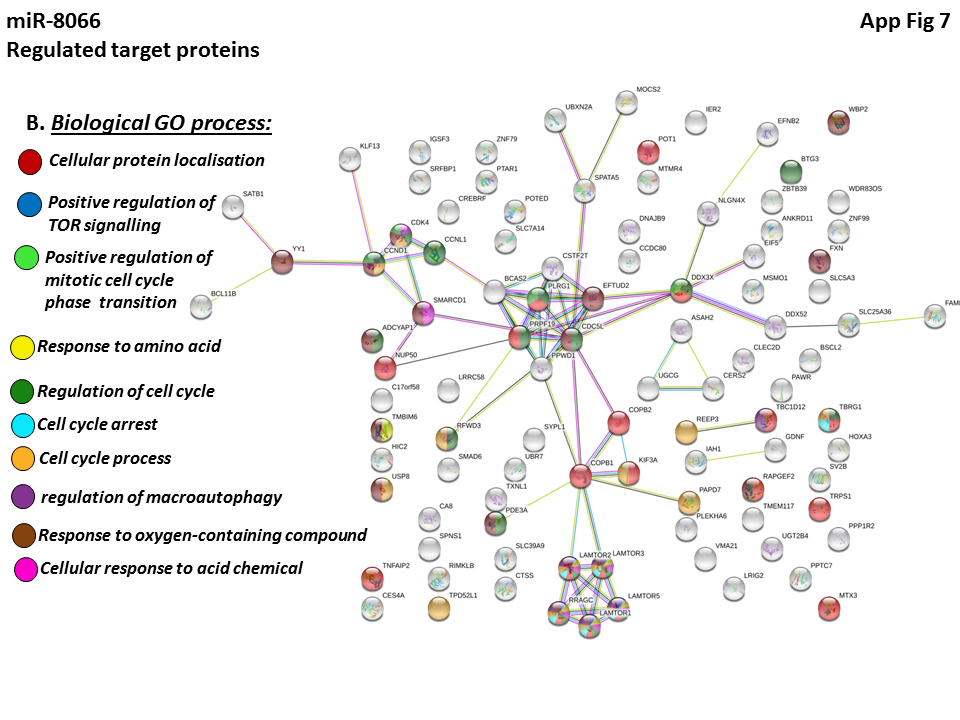

Supplement: Supplementary file 1 [file viruses-13-00117-s001.zip › Supp Fig S7_B_miR Zoonosis_2020.tif]

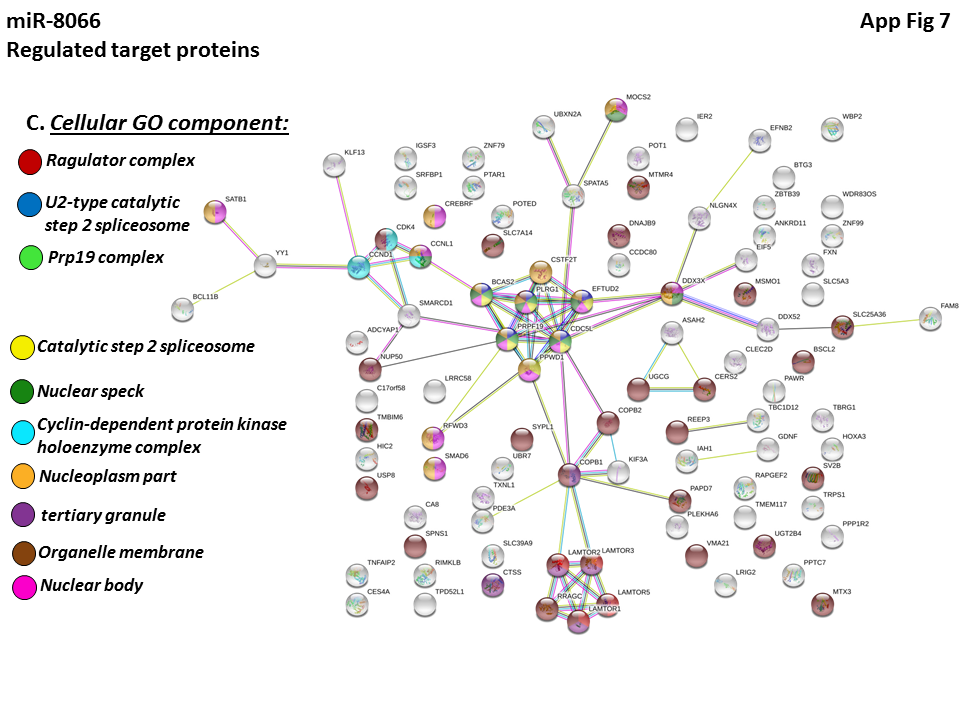

Supplement: Supplementary file 1 [file viruses-13-00117-s001.zip › Supp Fig S7_C_miR Zoonosis_2020.tif]
